# Anti-DFS70 antibodies in systemic lupus erythematosus: Prevalence in a large Chinese cohort and an unexpected association with anti-dsDNA antibodies by a long-term follow-up

**DOI:** 10.3389/fimmu.2022.913714

**Published:** 2022-09-14

**Authors:** Yingxin Dai, Enling Li, Dandan Chen, Xiangyu Niu, Zhiqing Wang, Liangjing Lu, Bing Zheng

**Affiliations:** ^1^ Department of Laboratory Medicine, Renji Hospital, Shanghai Jiao Tong University School of Medicine, Shanghai, China; ^2^ Department of Rheumatology, Renji Hospital, Shanghai Jiao Tong University School of Medicine, Shanghai, China

**Keywords:** autoantibodies, anti-DFS70, anti-dsDNA, follow-up, systemic lupus erythematosus

## Abstract

**Objective:**

Monospecific autoantibodies to dense fine speckles 70 (DFS70) antigen are purported to aid in excluding systemic autoimmune rheumatic diseases (SARD) such as systemic lupus erythematosus (SLE). However, the non-isolated anti-DFS70 still has a certain prevalence in SLE patients, and the clinical significance remains unclear. We aimed to investigate the prevalence, clinical relevance, and value of long-term monitoring of anti-DFS70 antibodies in SLE patients.

**Methods:**

Anti-DFS70 antibodies were measured by enzyme-linked immunosorbent assay (ELISA) in 851 SLE patients, 211 healthy individuals, and 194 patients with other SARD (except SLE). Demographic, serological, and clinical associations of anti-DFS70 antibodies were analyzed by a stepwise multivariable logistic regression model. The correlation of anti-DFS70 with anti-dsDNA, anti-C1q, and SLE Disease Activity Index 2000 (SLEDAI-2K) was analyzed. Sixty-one SLE patients with follow-up time ranging from 2 to 57 months were measured anti-DFS70 antibodies using both ELISA and line immunoassay. The dynamic variations of anti-DFS70 antibodies were evaluated with anti-dsDNA, anti-C1q, and SLEDAI-2K during the follow-up.

**Results:**

The prevalence of anti-DFS70 was significantly higher in SLE (20.7% (176/851)) than in healthy individuals (9.5% (20/211), *p* = 0.0002) and other SARD (10.8% (21/194), *p* = 0.002). Multivariable analysis revealed that anti-DFS70-positive SLE patients were associated with younger age (odds ratio (OR) = 0.982; 95% confidence interval (CI) = 0.969, 0.995), higher frequencies of anti-dsDNA (OR 1.598; 95% CI 1.107, 2.306) and anti-PCNA (OR 6.101; 95% CI 2.534, 14.688), and higher levels of serum IgG (OR 1.097; 95% CI 1.067, 1.129) and were more likely to be accompanied by mucosal ulcers (OR 5.921; 95% CI 1.652, 21.215). The O.D. value of anti-DFS70 positively correlated with levels of anti-dsDNA (*r* = 0.183, *p* < 0.0001) and anti-C1q (*r* = 0.181, *p* < 0.0001), respectively, but not with SLEDAI-2K (*p* = 0.920). During the follow-up, 49 (42 negative and 7 positive) patients remained stable with anti-DFS70 levels. The other 12 patients experienced significant changes in anti-DFS70, and 83.3% (10/12) of them showed similar trends between anti-DFS70 and anti-dsDNA by evaluation of dynamic variations.

**Conclusion:**

Anti-DFS70 antibodies seem to be prevalent in Chinese SLE patients. The positive association of anti-DFS70 with anti-dsDNA and consistent dynamic variation between anti-DFS70 and anti-dsDNA during the follow-up suggested a potential relationship between anti-DFS70 and anti-dsDNA in patients with SLE.

## Introduction

The dense fine speckles 70 (DFS70) antigen was a 70-kDa protein detected by immunoblotting, also known as the lens epithelium-derived growth factor (LEDGF) (1) and/or DNA binding transcription co-activator p75 (2). The DFS70/LEDGFp75 is a prosurvival factor that confers resistance to apoptosis induced by cell stress (1) and is also involved as a cofactor in HIV replication through an interaction with viral integrase (3). Anti-DFS70 antibodies, initially reported in a patient with interstitial cystitis in 1994, were later found in various conditions, such as chronic inflammatory diseases, cancers, and even in healthy individuals (4–6). It was reported that antinuclear antibodies (ANAs) can be positive in healthy individuals up to 20%, and the positivity in the majority of cases might be associated with anti-DFS70 antibodies (7). Although the presence of autoantibodies, including ANA, is a hallmark of systemic autoimmune rheumatic diseases (SARD), the isolated anti-DFS70 antibodies are considered by some rheumatologists as a tool to help exclude the diagnosis of SARD (7).

In the past few years, increasing concerns have already been raised regarding the prevalence and clinical significance of anti-DFS70 antibodies in SARD cohorts. A recent study suggested anti-DFS70 antibodies were prevalent in connective tissue disease (CTD) patients, while the monospecific anti-DFS70 antibodies were rare (8). In systemic lupus erythematosus (SLE) patients, the positive rates of anti-DFS70 ranged from 0% to 22.1% by different methodologies among several studies (9–13). The majority of them regarded the anti-DFS70 antibodies as less prevalent in SLE patients than in healthy individuals, except for one research performed by Japanese scholars, indicating that there was no significant difference in the prevalence of anti-DFS70 antibodies between SLE patients (22.1%) and healthy individuals (16.4%) (11). The diversity of the prevalence of anti-DFS70 antibodies may be affected by genetic, ethnic, and environmental factors, as well as detection methods. To date, few studies have investigated the anti-DFS70 antibodies in SLE patients in China (14, 15).

As for the clinical significance of anti-DFS70 antibodies, there are still different opinions. Mahler et al. found no clinical or laboratory differences between anti-DFS70-positive and anti-DFS70-negative SLE patients (12). Aragón et al. reported the negative association of anti-dsDNA antibodies, erythrocyte sedimentation rate (ESR), and the positive correlation of complement 3 (C3) levels with anti-DFS70 antibodies (16). One multicenter research reported that SLE patients with musculoskeletal activity and anti-β2 glycoprotein 1–positive patients were more likely to have anti-DFS70 antibodies, while those with anti-dsDNA, anti-SSA/Ro60, anti-SSB/La, or anti-U1-RNP antibodies were less likely to have anti-DFS70 antibodies (9). Chen et al. reported that anti-DFS70 antibodies were more common in proliferative lupus nephritis (PLN) than in membrane lupus nephritis (MLN), and the optical density O.D. value by enzyme-linked immunosorbent assay (ELISA) was associated with renal pathological activity index, highlighting the importance of investigating anti-DFS70 clinical relevance in SLE patients (15).

Given that the role of the anti-DFS70 antibodies in SLE patients has not been fully explored, further investigations are essential for studying the clinical relevance of anti-DFS70 antibodies in SLE patients. In addition, to our knowledge, no research has investigated the variations of the levels of anti-DFS70 antibodies in long-term follow-up SLE cohorts, which are vital to a better understanding of the role of anti-DFS70 antibodies in SLE.

Our study aimed to investigate the prevalence of anti-DFS70 antibodies in the Chinese SLE cohort versus disease control and age- and gender-matched healthy control groups using ELISA. We also compared the demographic, serological, and clinical features between anti-DFS70-positive and anti-DFS70-negative SLE patients; analyzed the correlation between anti-DFS70 and markers of disease activity; and evaluated the variations of anti-DFS70 antibody levels by a long-term follow-up.

## Materials and methods

### Study population

#### Disease group

A total of 851 adult SLE inpatients who were admitted to Renji Hospital (Shanghai, China) from June 2016 to December 2018 were enrolled. All patients fulfilled the revised classification criteria for SLE of the American College of Rheumatology (17). SLE patients who were complicated by other SARD or malignancies were excluded.

#### Control groups

The healthy control (HC) cohort included 211 age- and gender-matched healthy individuals from the physical examination center, without any known history of SARD or chronic diseases. The disease control (DC) cohort enrolled 194 SARD patients (except for SLE) who went to Renji Hospital during the same period as the SLE cohort and consisted of 32 Sjögren syndrome (SS), 34 mixed connective tissue disease (MCTD), 44 dermatomyositis/polymyositis (DM/PM), 73 rheumatoid arthritis (RA), and 11 scleroderma patients.

#### Follow-up group

A total of 61 patients from the SLE cohort with two or more medical records from June 2016 to April 2021 were enrolled as follow-up patients.

Patient demographic and clinical data were collected, including age, gender, disease duration, disease activity, clinical features, and symptoms, as well as the use of medications. The disease activity of SLE was evaluated according to the Systemic Lupus Erythematosus Disease Activity Index 2000 (SLEDAI-2K) (18).

All samples were collected from clinical residual specimens, and the study was approved by the Institutional Review Board of Renji Hospital. No informed consent was required for this study.

### Anti-DFS70 antibodies detection

Anti-DFS70 antibodies were detected by ELISA, as described in our previous study (14). Briefly, 0.5 μg/ml purified recombinant DFS70 antigen (DIARECT AG, Freiburg, Germany) was coated in 96-well plates overnight at 4°C. After blocking, patient sera were diluted 1:200 in serum diluent and added to each well for 2 h of incubation at room temperature with moderate shaking. The horseradish peroxidase-conjugated AffiniPure rabbit anti-human IgG (Jackson ImmunoResearch, West Grove, PA) as the secondary antibody was diluted 1:10,000 in an anti-immunoglobulin diluent and also incubated for 2 h at room temperature with moderate shaking. After that, samples were developed using 3,3′,5,5′-tetramethylbenzidine (TMB, EUROIMMUN, Lübeck, Germany), and the O.D. value was read at 450 nm by a microplate reader (Multiskan FC, Thermo Fisher, Waltham, MA, USA).

The presence of anti-DFS70 antibodies in 149 samples of 61 follow-up patients was also tested by line immunoassay (LIA), as previously described (14), using an IMTEC-ANA-LIA XL Assay kit (HUMAN Diagnostics Worldwide, Wiesbaden, Germany). A HumanScan system was used to analyze and interpret the results according to the manufacturer’s instructions. Classification of line intensity by LIA was conducted as follows: index 0–0.79, intensity (−), negative; index 0.8–1.14, intensity (o), borderline; index 1.15–2.49, intensity (+), weak positive; index 2.50–3.99, intensity (++), mid-level positive; and index ≥4.00, intensity (+++), strong positive. Anti-DFS70 reference serum (19) was used as the positive control for both assays.

### Testing of autoantibodies

The quantitative determination of anti-dsDNA (Trinity Biotech plc, Wicklow, Ireland) and anti-C1q (EUROIMMUN) antibodies was performed by ELISA using the Sprinter XL automated IFT/ELISA Analyzer (EUROIMMUN), as well as the qualitative detection of antinucleosome (EUROIMMUN) and anticardiolipin (EUROIMMUN) antibodies. Autoantibodies to Sm, nRNP/Sm, Ro52, SSA/Ro60, SSB/La, proliferating cell nuclear antigen (PCNA), and ribosomal-P (Rib-p) were measured by LIA using a EUROLineMaster Plus automated LIA Analyzer (EUROIMMUN). All assays were performed according to the manufacturer’s instructions.

### Other laboratory examinations

Other laboratory examinations included C3, complement 4 (C4), C-reactive protein (CRP), ESR, serum immunoglobulin A (IgA), IgG, and IgM. The levels of C3, C4, IgA, IgG, and IgM were measured by immunonephelometry (Siemens Healthcare Diagnostics Inc., Newark, USA). In addition, CRP was determined by a BC-5390 CRP Auto Hematology Analyzer (Mindray Co. Ltd., Shenzhen, China). ESR was tested by an Automated ESR Analyzer (Vital Diagnostics S.r.l., Forli, Italy).

### DNA adsorption

To determine whether the anti-DFS70 reactivity results from anti-DNA, the DNA adsorption was performed on three classes of serum sampling from the SLE cohort, including six anti-DFS70/anti-dsDNA dual positive serum samples, four anti-DFS70 (−)/anti-dsDNA (+) serum samples, and three anti-DFS70 (+)/anti-dsDNA (−) serum samples. All these samples were diluted in 1:200 in serum diluent and divided into two equal parts. One part was for the DNA absorption test, which was treated with 30 µg/ml of UltraPure Herring Sperm DNA (Thermo Fisher Scientific, Carlsbad,USA) and incubated at room temperature for 1 h with moderate shaking. The other equality was not added with DNA and was kept in the same condition as the control group. After incubation, all the serum samples were determined to contain anti-DFS70 antibodies by ELISA as described above and tested for anti-dsDNA antibodies using a commercial ELISA kit (EUROIMMUN) according to the manufacturer’s instructions.

### Statistical analysis

Statistical analysis was performed using SPSS 23.0 software (IBM-SPSS, Inc., Armonk, NY, USA). Normally and non-normally distributed continuous variables were respectively represented by mean ± standard deviation (SD) and median with interquartile range (IQR). Categorical variables were expressed as counts and percentages. The two-tailed chi-square (*χ* ^2^) test or Fisher’s exact test was carried out to analyze the differences in the prevalence of anti-DFS70 antibodies between the two groups and the differences in the use of medications in follow-up patients between the first and last visits. A stepwise multivariable logistic regression analysis was used to identify covariates associated with positive anti-DFS70 antibodies in demographic, serological, and clinical features. Covariates with a *p*-value of <0.05 in the univariable logistic regression analysis were incorporated into the multivariable model. The results were expressed as an odds ratio (OR) with a 95% confidence interval (CI). The Wilcoxon matched-pairs tests were performed to compare the SLEDAI-2K, anti-dsDNA titers, anti-C1q titers, and prednisone dose of follow-up patients between the first and last visits. GraphPad Prism 6.0 software (GraphPad Software Inc., La Jolla, USA) was used for drawing charts. The Spearman’s rank correlation test was used to assess the relationship between anti-DFS70 and markers of disease activity (anti-dsDNA, anti-C1q, and SLEDAI-2K). *p* < 0.05 was considered statistically significant.

For the evaluation of the long-term monitoring value of anti-DFS70 titers, SLE patients in the follow-up group were defined into different categories as follows: the anti-DFS70 changed group represented that the levels of anti-DFS70 significantly changed, including the conversion between anti-DFS70-positive and anti-DFS70-negative results, as well as one or more levels’ change of positive results within the intensity classification scheme of LIA; the anti-DFS70 stable group indicated that the levels of anti-DFS70 remained stable during the follow-up, including remaining at the same level, and changed between the negative result and borderline or changed between a weak positive result and borderline.

## Results

### Patients’ characteristics

The characteristics of 851 SLE patients from the disease group, 211 healthy individuals, and 194 SARD patients from control groups, as well as 61 SLE patients from the follow-up group, are summarized in **Table 1**. Patients in the long-term follow-up group have a median (range) follow-up time of 11.0 (2.0–57.0) months with an average visit frequency of 2.4 times.

**Table 1:** Patient characteristics of the SLE cohort, SLE follow-up patients, health control, and disease control.

	SLE		SLE follow-up	HC	DC
Number of patients	851		61	211	194
Sex [*n* (%)]					
Women	776 (91.2)		54 (88.5)	192 (91.0)	186 (95.9)
Men	75 (8.8)		7 (11.5)	19 (9.0)	8 (4.1)
Age (mean ± SD; years)	40.1 ± 13.8		41.89 ± 13.53^a^	40.6 ± 13.6	41.4 ± 12.8
Disease duration of SLE [median (IQR); years]	5.0 (1.0–10.0)		4.0 (1.7–9.5)^a^	N/A	N/A
SLEDAI-2K [median (IQR)]	8.0 (4.0–12.0)		6.0 (2.0–9.0)^a^	N/A	N/A

DC, disease control; HC, healthy control; IQR, interquartile range; N/A, not applicable; SD, standard deviation; SLE, systemic lupus erythematosus; SLEDAI-2K, Systemic Lupus Erythematosus Disease Activity Index 2000.

a Data of the follow-up group at the time of enrollment.

### Prevalence of anti-DFS70 antibodies in different cohorts

In SLE patients, the prevalence of anti-DFS70 antibodies was 20.7% (176/851), which was significantly higher than HC (9.5% (20/211), *p* = 0.0002) and DC (10.8% (21/194), *p* = 0.002). Additionally, the positive rates of anti-DFS70 in MCTD, RA, SS, scleroderma, and DM/PM patients were 14.7% (5/34), 12.3% (9/73), 9.4% (3/32), 9.1% (1/11), and 6.8% (3/44), respectively. There was no significant difference in the rate of anti-DFS70-positive patients between DC and HC (DC (10.8%) *vs*. HC (9.5%), *p* = 0.654). The prevalence of “monospecific” anti-DFS70 antibodies (anti-dsDNA and other detected anti-extractable nuclear antigen negative) in SLE patients was 1.2% (10/851). Information on the 10 SLE patients with isolated anti-DFS70 antibodies is summarized in **Supplementary Table S1**. There was no significant difference in the prevalence of anti-DFS70 antibodies between male and female cases in SLE (men 14.7% (11/75) *vs.* women 21.3% (165/776), *p* = 0.178) and HC (men 15.8% (3/19) *vs.* women 8.9% (17/192), *p* = 0.566) cohorts.

### Demographic, serological, and clinical associations of anti-DFS70 antibodies

**Figure 1:**
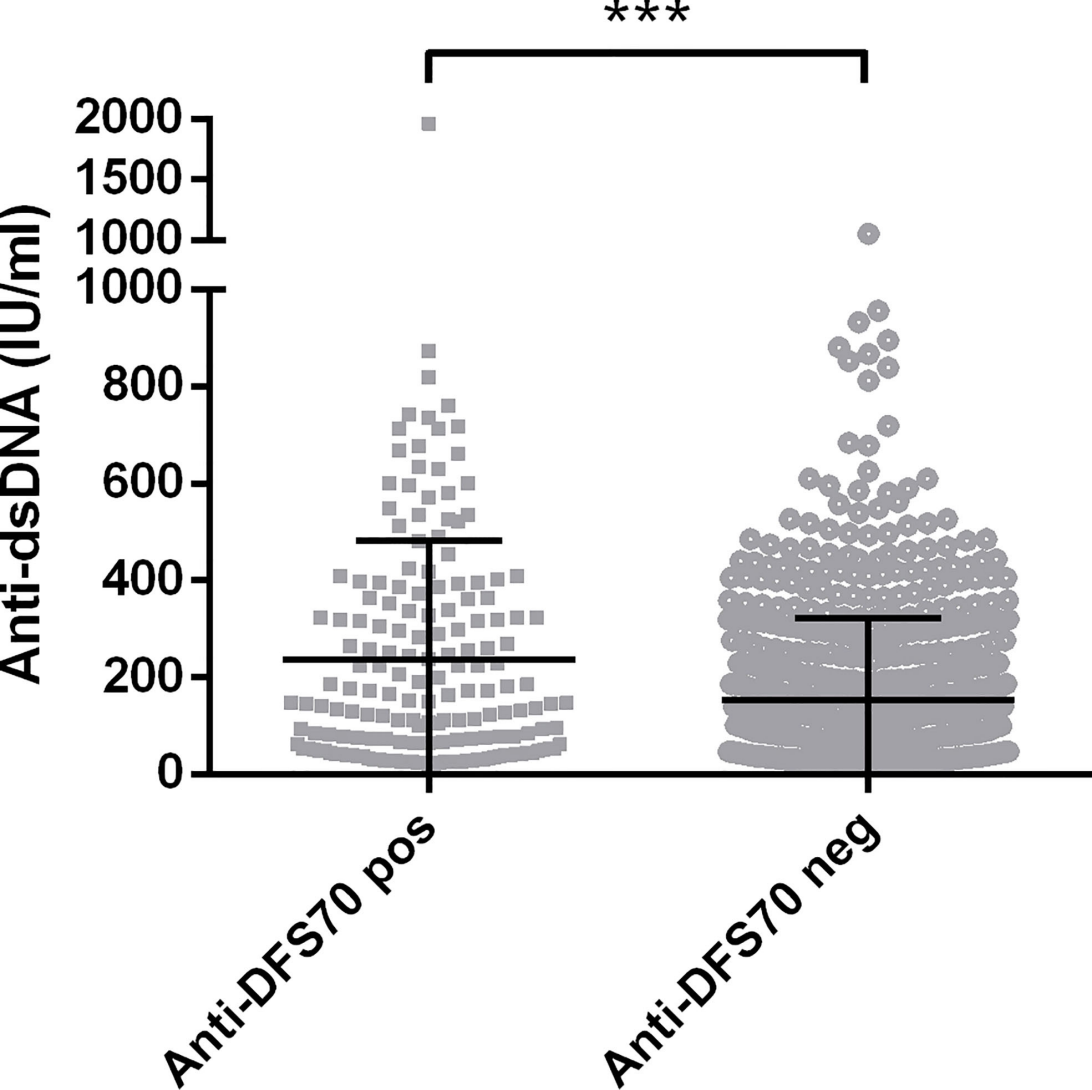
Comparison of levels of anti-dsDNA antibodies in 851 SLE patients with and without anti-DFS70 antibodies. Anti-DFS70 and anti-dsDNA antibodies were both measured by ELISA. DFS70, dense fine speckles 70; ELISA, enzyme-linked immunosorbent assay; neg, negative; pos, positive; SLE, systemic lupus erythematosus. ^***^ *p* < 0.001.

Comparisons of demographic, serological, medications, and clinical features between anti-DFS70-positive and anti-DFS70-negative SLE patients are shown in **Table 2** and **Supplementary Table S2**. According to the univariable logistic regression analysis, there were no significant differences in sex, disease duration, SLEDAI-2K, and use of immunosuppressive medications between anti-DFS70-positive and anti-DFS70-negative patients in the SLE cohort. However, it revealed significant associations of anti-DFS70-positive SLE patients with younger age, higher frequency of anti-dsDNA and anti-C1q antibodies, more rapid ESR, higher concentrations of serum IgA and IgG, and lower concentrations of C3 and C4 (**Table 2**). The anti-DFS70-positive patients also had a higher frequency of anti-nRNP/Sm and anti-PCNA, and they were more likely to be accompanied by mucosal ulcers and leukopenia (**Supplementary Table S2**). These covariates with significant differences were included in stepwise multivariable analysis. After adjustment, younger age, higher frequency of anti-dsDNA and anti-PCNA, and higher levels of serum IgG were still positively associated with the anti-DFS70 antibodies (**Table 2**). In the multivariable analysis of clinical features, mucosal ulcers also showed significant associations with anti-DFS70 antibodies (**Supplementary Table S2**). Moreover, we plotted the distribution of anti-dsDNA titers between anti-DFS70-positive and anti-DFS70-negative SLE patients in **Figure 1**, which showed significantly higher levels of anti-dsDNA in anti-DFS70-positive patients than anti-DFS70-negative patients (*p* < 0.0001). 

**Table 2:** Comparison of demographic and serological parameters between anti-DFS70-positive and anti-DFS70-negative SLE patients.

Characteristics	Anti-DFS70 positive [*n* = 176; *n* (%)]	Anti-DFS70 negative [*n* = 675; *n* (%)]	Univariable analysis		Multivariable analysis	
			Unadjusted OR (95% CI)	*p*-value	Adjusted OR (95% CI)	*p*-value
Age (years)^a^	38.0 ± 12.7	40.7 ± 14.0	0.985 (0.973, 0.998)	**0.022**	0.982 (0.969, 0.995)	**0.007**
Sex (women)	165 (93.8)	611 (90.5)	1.571 (0.810, 3.047)	0.181		
Duration (years)^b^	4.0 (0.9–10.0)	5.0 (1.0–10.0)	0.996 (0.972, 1.020)	0.713		
SLEDAI-2K^b^	8.0 (4.0–11.8)	7.0 (4.0–12.0)	1.018 (0.989, 1.048)	0.219		
Anti-dsDNA	117 (66.5)	326 (48.3)	2.123 (1.500, 3.005)	**<0.0001**	1.598 (1.107–2.306)	**0.012**
Anti-C1q	60 (34.1)	150 (22.2)	1.810 (1.262, 2.597)	**0.001**		
Anti-Sm	33 (18.8)	99 (14.7)	1.343 (0.870, 2.073)	0.184		
Anti-nRNP/Sm	81 (46.0)	245 (36.3)	1.496 (1.070, 2.093)	**0.018**		
Anti-Ro52	102 (58.0)	338 (50.1)	1.374 (0.983, 1.922)	0.063		
Anti-SSA/Ro60	104 (59.1)	367 (54.4)	1.212 (0.866, 1.697)	0.262		
Anti-SSB/La	23 (13.7)	76 (11.3)	1.185 (0.719, 1.952)	0.505		
Anti-PCNA	14 (8.0)	10 (1.5)	5.747 (2.507, 13.173)	**<0.0001**	6.101 (2.534, 14.688)	**<0.0001**
Anti-Rib-P	44 (25.0)	143 (21.2)	1.240 (0.841, 1.828)	0.277		
Anticardiolipin	9 (5.1)	49 (7.3)	0.689 (0.331, 1.430)	0.317		
C3^a^ (g/L)	0.7 ± 0.3	0.8 ± 0.3	0.358 (0.201, 0.638)	**0.0005**		
C4^a^ (g/L)	0.1 ± 0.1	0.1 ± 0.1	0.015 (0.002, 0.143)	**0.0002**		
CRP^b^ (mg/L)	3.3 (3.1–9.1)	3.2 (2.9–8.5)	0.996 (0.988, 1.005)	0.389		
ESR^a^ (mm/h)	43.7 ± 33.0	36.2 ± 28.5	1.008 (1.003, 1.013)	**0.003**		
Serum IgA^a^ (g/L)	2.9 ± 1.4	2.6 ± 1.3	1.166 (1.039, 1.308)	**0.009**		
Serum IgG^a^ (g/L)	17.0 ± 7.6	13.1 ± 5.6	1.095 (1.066, 1.125)	**<0.0001**	1.097 (1.067, 1.129)	**<0.0001**
Serum IgM^a^ (g/L)	1.1 ± 0.7	1.0 ± 0.7	1.202 (0.962, 1.502)	0.105		
Prednisone dose^b^ (mg)	30 (15–60)	30 (12.5–40)	1.002 (0.999, 1.006)	0.203		
Hydroxychloroquine	111 (63.1)	411 (60.9)	1.097 (0.778, 1.546)	0.597		
Mycophenolate mofetil	21 (11.9)	101 (15.0)	0.770 (0.466, 1.273)	0.308		
Cyclophosphamide	10 (5.7)	45 (6.7)	0.843 (0.416, 1.709)	0.636		
Tacrolimus	8 (4.5)	33 (4.9)	0.926 (0.420, 2.043)	0.850		
Cyclosporin A	7 (4.0)	22 (3.3)	1.229 (0.517, 2.926)	0.641		
Azathioprine	2 (1.1)	18 (2.7)	0.420 (0.096, 1.825)	0.247		
No immunosuppressants at present	38 (21.6)	157 (23.3)	0.909 (0.608, 1.357)	0.639		

p < 0.05 is shown in bold.

C3, complement 3; C4, complement 4; CRP, C-reactive protein; DFS70, dense fine speckles 70; ds-DNA, double-stranded DNA; ESR, erythrocyte sedimentation rate; OR, odds ratio; PCNA, proliferating cell nuclear antigen; Rib-P, ribosomal-P; SLE, systemic lupus erythematosus; SLEDAI-2K, Systemic Lupus Erythematosus Disease Activity Index 2000.

a Average ± standard deviation.

b Median (interquartile range).

### Correlations of anti-DFS70 with markers of disease activity in both SLE cohort and follow-up group

**Figure 2:**
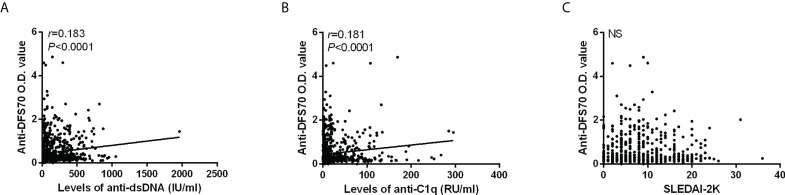
Correlations of anti-DFS70 O.D. value by ELISA with markers of disease activity in 851 SLE patients. Correlation of O.D. value of anti-DFS70 antibodies with the levels of anti-dsDNA antibodies **(A)**, anti-C1q antibodies **(B)**, and SLEDAI-2K **(C)**. DFS70, dense fine speckles 70; ELISA, enzyme-linked immunosorbent assay; NS, no significance; O.D. value, optical density value; *r*, correlation coefficient; SLEDAI-2K, Systemic Lupus Erythematosus Disease Activity Index 2000.

For 851 patients in the SLE cohort, we performed Spearman’s rank correlation analysis to evaluate the correlations of anti-DFS70 antibodies with some markers of disease activity. **Figure 2** shows the O.D. value of anti-DFS70 positively correlated with the levels of anti-dsDNA (*r* = 0.183, *p* < 0.0001) and anti-C1q (*r* = 0.181, *p* < 0.0001), while no significant correlation with SLEDAI-2K was observed (*p* = 0.920).

**Figure 3:**
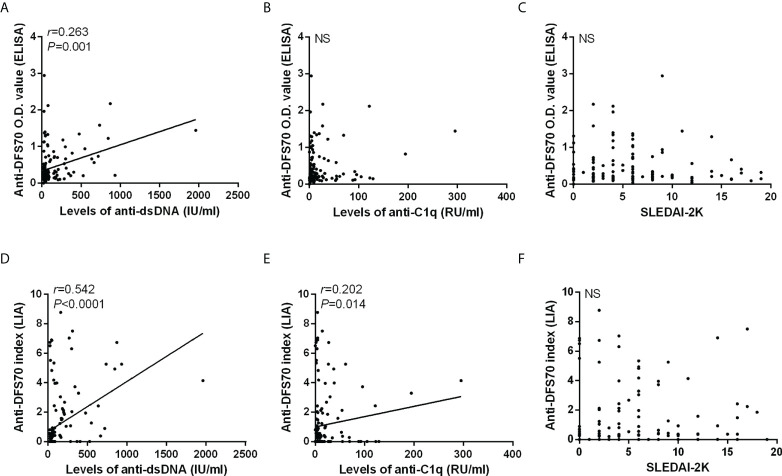
Correlations of anti-DFS70 with markers of disease activity in 149 sera from 61 follow-up SLE patients. Correlations of anti-DFS70 O.D. value by ELISA with the levels of anti-dsDNA antibodies **(A)**, anti-C1q antibodies **(B)**, and SLEDAI-2K **(C)**. Correlations of the anti-DFS70 index by LIA with the levels of anti-dsDNA antibodies **(D)**, anti-C1q antibodies **(E)**, and SLEDAI-2K **(F)**. DFS70, dense fine speckles 70; ELISA, enzyme-linked immunosorbent assay; LIA, line immunoassay; NS, no significance; O.D. value, optical density value; *r*, correlation coefficient; SLEDAI-2K, Systemic Lupus Erythematosus Disease Activity Index 2000.

Additionally, we also carried out a correlation analysis of anti-DFS70 antibodies with anti-dsDNA, anti-C1q, and SLEDAI-2K in 149 serum samples from 61 follow-up patients using the O.D. value of anti-DFS70 by ELISA (**Figures 3A–C**) and the anti-DFS70 index by LIA (**Figures 3D–F**). The positive correlations were observed both in the O.D. value of anti-DFS70 with levels of anti-dsDNA (*r* = 0.263, *p* = 0.001) and the anti-DFS70 index by LIA with levels of anti-dsDNA (*r* = 0.542, *p* < 0.0001) and anti-C1q (*r* = 0.202, *p* = 0.014). On the other hand, no significant correlation was shown between either the O.D. value (*p* = 0.930) or the index (*p* = 0.326) of anti-DFS70 and SLEDAI-2K.

### Characteristics of patients and variations of anti-DFS70 antibodies with markers of disease activity in the follow-up group

In the follow-up group, 149 serum samples obtained from 61 SLE patients were measured for anti-DFS70 antibodies using both ELISA and LIA. The O.D. values of anti-DFS70 by ELISA were positively associated with the anti-DFS70 index by LIA (*r* = 0.566, *p* < 0.0001), and the coincidence rate between the two methods was 87.2% (130/149) (**Supplementary Figure S1**). According to the classification of line intensity by LIA, SLE follow-up patients were grouped by variations of anti-DFS70 antibodies into anti-DFS70 stable and changed groups. The anti-DFS70 stable group included 49 patients who remained in stable anti-DFS70 levels during the follow-up, in which 42 remained negative and 7 remained positive. The remaining 12 patients who experienced significant changes in anti-DFS70 antibodies belonged to the anti-DFS70 changed group, including 8 with decreasing results and 4 with increasing results of anti-DFS70 antibodies from the first visit to the last visit.

The differences in demographic features, markers of disease activity, and medications of SLE follow-up patients were compared between the first and last visits in **Table 3**. In the anti-DFS70 stable group, no significant differences were observed in indicators such as markers of disease activity and types of immunosuppressants, except that patients who maintained negative anti-DFS70 antibodies during the follow-up received decreased prednisone dose at the last visit compared with the first visit. Meanwhile, in anti-DFS70 changed groups (**Table 3**), patients with decreasing trend of anti-DFS70 antibodies from the first visit to the last visit showed significantly decreased levels of anti-dsDNA at the same period (*p* = 0.012). Patients who experienced increasing results of anti-DFS70 antibodies during the follow-up showed a similar change of anti-dsDNA titers as well, with no statistical significance (*p* = 0.144).

**Table 3:** Characteristics of follow-up patients with SLE as grouped by variation of anti-DFS70 antibodies at the first and last visits.

	Anti-DFS70 stable, negative group (*n* = 42)			Anti-DFS70 stable, positive group (*n* = 7)			Anti-DFS70 changed, decreasing group (*n* = 8)			Anti-DFS70 changed, increasing group (*n* = 4)		
	First visit	Last visit	*p*-value	First visit	Last visit	*p*-value	First visit	Last visit	*p*-value	First visit	Last visit	*p*-value
Age (years)^a^	42.9 ± 13.6	43.9 ± 13.5	N/A	31.0 ± 13.8	35.1 ± 14.2	N/A	42.4 ± 9.2	44.0 ± 9.7	N/A	49.8 ± 13.1	50.8 ± 13.6	N/A
Sex (men/women)	5/37	5/37	N/A	0/7	0/7	N/A	1/7	1/7	N/A	3/1	3/1	N/A
Duration (years)^b^	4.0 (1.0–8.3)	4.5 (2.4–10.3)	N/A	12.0 (7.0–12.0)	16.4 (8.4–16.7)	N/A	6.5 (1.3–13.3)	7.0 (3.4–17.3)	N/A	2.7 (1.0–4.8)	3.3 (2.1–6.1)	N/A
Follow-up time (months)^b^	N/A	8.5 (5.8–18.0)	N/A	N/A	55 (46–57)	N/A	N/A	11.0 (4.0–36.3)	N/A	N/A	12.0 (6.5–18.3)	N/A
SLEDAI-2K^b^ ^,^ ^d^	5.0 (2.0–8.3)	4.0 (1.5–8.0)	0.104	4.0 (2.0–9.0)	4.0 (2.0–6.0)	0.246	10.0 (6.5–16.0)	10.0 (6.5–15.5)	0.547	2.0 (2.0–5.8)	5.5 (2.8–14.3)	0.109
Anti-dsDNA (IU/ml)^b^ ^,^ ^d^	37.3 (26.3–51.7)	37.4 (24.8–63.5)	0.440	199.0 (45.0–548.1)	160.9 (48.0–475.9)	0.612	363.5 (197.5–857.8)	83.5 (75.9–164.7)	**0.012**	80.6 (42.5–300.3)	193.2 (157.4–284.3)	0.144
Anti-C1q (RU/ml)^b^ ^,^ ^d^	5.5 (3.4–10.5)	4.6 (1.6–10.4)	0.120	5.2 (3.5–26.6)	5.8 (4.1–29.9)	0.398	43.1 (6.7–169.7)	50.5 (3.8–86.4)	0.263	29.3 (2.4–110.3)	13.8 (5.0–20.6)	0.465
Prednisone dose (mg)^b^ ^,^ ^d^	15.0 (10.0–30.0)	10.0 (10.0–13.1)	**0.001**	10.0 (10.0–10.0)	7.5 (7.5–10.0)	0.059	35.0 (22.5–87.5.0)	25.0 (16.3–52.5)	0.207	17.5 (5.0–45)	20.0 (8.1–82.5)	0.273
Hydroxychloroquine^c^ ^,^ ^e^	31 (73.8)	33 (78.6)	0.608	6 (85.7)	6 (85.7)	1.000	5 (62.5)	4 (50.0)	1.000	2 (50.0)	0 (0.0)	0.429
Mycophenolate mofetil^c^ ^,^ ^e^	12 (28.6)	14 (33.3)	0.637	2 (28.6)	1 (14.3)	1.000	2 (25.0)	3 (37.5)	1.000	2 (50.0)	2 (50.0)	1.000
Cyclophosphamide^c^ ^,^ ^e^	14 (33.3)	13 (31.0)	0.815	1 (14.3)	0 (0.0)	1.000	0 (0.0)	3 (37.5)	0.200	0 (0.0)	1 (25.0)	1.000
Tacrolimus^c^ ^,^ ^e^	3 (7.1)	3 (7.1)	1.000	1 (14.3)	1 (14.3)	1.000	1 (12.5)	1 (12.5)	1.000	0 (0.0)	0 (0.0)	N/A
Cyclosporin A^c^ ^,^ ^e^	2 (4.8)	0 (0.0)	0.474	0 (0.0)	0 (0.0)	N/A	0 (0.0)	0 (0.0)	N/A	0 (0.0)	0 (0.0)	N/A
Azathioprine^c^ ^,^ ^e^	1 (2.4)	6 (14.3)	0.114	1 (14.3)	2 (28.6)	1.000	1 (12.5)	0 (0.0)	1.000	0 (0.0)	0 (0.0)	N/A
No immunosuppressants used^c^ ^,^ ^e^	2 (4.8)	2 (4.8)	1.000	0 (0.0)	1 (14.3)	1.000	0 (0.0)	1 (12.5)	1.000	1 (25.0)	1 (25.0)	1.000

Anti-DFS70 changed represented that the levels of anti-DFS70 significantly changed, including the conversion between positive and negative, and changed one or more levels of positive results within the intensity classification scheme of LIA, and according to the change trend by comparing results of anti-DFS70 by LIA at the last visit to the first visit during the follow-up, and the patients were divided into the decreasing and increasing groups. Anti-DFS70 stable indicated that the levels of anti-DFS70 remained stable during the follow-up, including remaining at the same level or changing between negative and borderline or changing between weak positive and borderline; by the positive and negative results of DFS70 by LIA, those patients were divided into the positive and negative groups, separately. Classification of line intensity by LIA: index 0–0.79, intensity (−), negative; index 0.8–1.14; intensity (o), borderline; index 1.15–2.49, intensity (+), weak positive; index 2.50–3.99, intensity (++), mid-level positive; and index ≥4.00, intensity (+++), strong positive. p-value defines differences between inclusion (first visit) and follow-up (last visit); p < 0.05 is shown in bold.

N/A, not applicable; DFS70, dense fine speckles 70; ds-DNA, double-stranded DNA; SLE, systemic lupus erythematosus; SLEDAI-2K, Systemic Lupus Erythematosus Disease Activity Index 2000.

a Average ± standard deviation.

b Median (interquartile range).

c Numbers (%).

d Wilcoxon matched-pairs test.

e The two-tailed chi-square (χ^2^) test or Fisher’s exact test.

**Figure 4:**
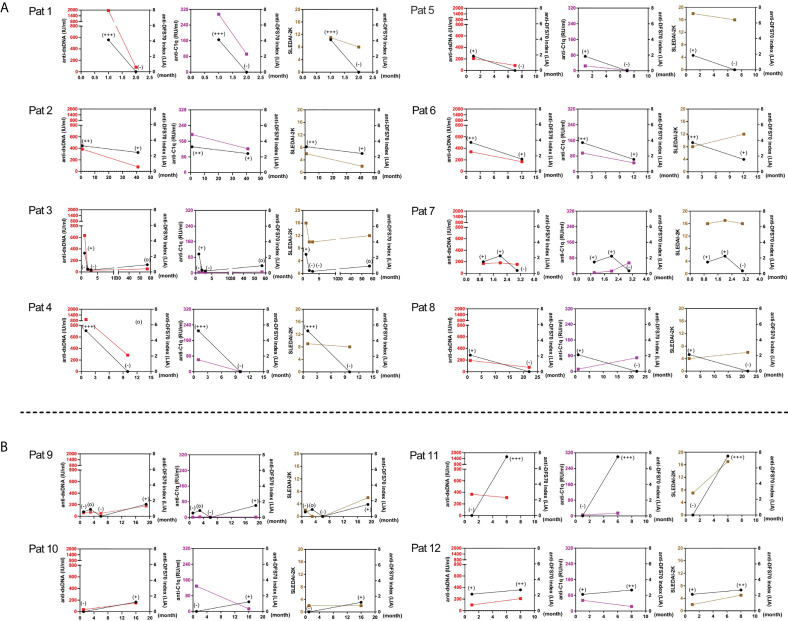
Variations of the levels of anti-dsDNA, anti-C1q antibodies, and SLEDAI-2K in 12 SLE follow-up patients having significant changes of anti-DFS70 antibodies by LIA. Significant changes in the levels of anti-DFS70 antibodies, including the conversion between positive and negative, and changed one or more positive levels within the intensity classification scheme of LIA. The upper and lower panels respectively show patients with decreasing and increasing results of anti-DFS70 antibodies by LIA from the first visit to the last visit during the follow-up. **(A)** In patients with a downtrend of anti-DFS70 antibodies (Pat 1-8), a similar variation trend was observed between anti-DFS70 and anti-dsDNA in seven of eight patients except for Pat 7. A similar variation trend was observed between anti-DFS70 and anti-C1q in five of eight patients except for Pat 3, Pat 7, and Pat 8. A similar variation trend was observed between anti-DFS70 and SLEDAI-2K in four of eight patients except for Pat 4, Pat 6, Pat 7, and Pat 8. **(B)** In patients with an upward trend of anti-DFS70 antibodies (Pat 9–12), a similar variation trend was observed between anti-DFS70 and anti-dsDNA in three of four patients except for Pat 11. No similar variation trend was observed between anti-DFS70 and anti-C1q. A similar variation trend was observed between anti-DFS70 and SLEDAI-2K in three of four patients, except for Pat 10. Red, anti-dsDNA; purple, anti-C1q; brown, SLEDAI-2K; black, anti-DFS70; *x*-axis, time points of measurement (the time point of the first visit was set as 1). Classification of line intensity by LIA: index 0–0.79, intensity (−), negative; index 0.8–1.14; intensity (o), borderline; index 1.15–2.49, intensity (+), weak positive; index 2.50–3.99, intensity (++), mid-level positive; index ≥4.00, intensity (+++), strong positive. DFS70, dense fine speckles 70; LIA, line immunoassay; Pat, patient; SLEDAI-2K, Systemic Lupus Erythematosus Disease Activity Index 2000.

To assess the dynamic change of anti-DFS70 antibodies with markers of disease activity during the follow-up, we summarized 12 patients with significant changes in the levels of anti-DFS70 antibodies in **Figure 4**, which presents patients who experienced decreasing results (**Figure 4A**) and increasing results (**Figure 4B**) of anti-DFS70 antibodies during the follow-up, respectively. A consistent variation trend between anti-DFS70 and anti-dsDNA was observed in 83.3% (10/12) of them, except for Pat 7 and Pat 11. Moreover, similar variation trends were also observed between anti-DFS70 and anti-C1q in 41.7% (5/12) of them and between anti-DFS70 and SLEDAI-2K in 58.3% (7/12) of these patients.

### DNA adsorption test

Anti-DFS70 reactivity has no relevance to the existence of anti-DNA antibodies in sera. The titers of anti-dsDNA antibodies in all six anti-DFS70 (+)/anti-dsDNA (+) serum samples were significantly decreased after DNA adsorption compared with the untreated control group, while their anti-DFS70 O.D. values remained stable (**Supplementary Table S3**).

## Discussion

We conducted an investigation of anti-DFS70 antibodies in a large SLE cohort and other SARD disease controls as well as an age- and gender-matched HC group in Chinese. To our knowledge, the present study was the first to assess the changes in anti-DFS70 antibodies in SLE patients through a long-term follow-up. Several previous studies have indicated a protective role of isolated anti-DFS70 antibodies. A 4-year follow-up study reported that healthy individuals with isolated anti-DFS70 reactivity were less likely to progress to SARD (20). The prevalence of monospecific anti-DFS70 antibodies was significantly higher in patients with non-ANA-associated rheumatic diseases (AARD) and undifferentiated CTD (UCTD) than in patients with AARD (21). UCTD patients with monospecific anti-DFS70 antibodies were at low risk of progression to CTD (22). Although the monospecific anti-DFS70 antibodies are more predominant in healthy individuals and have been proposed as a biomarker for the exclusion of SARD, the anti-DFS70 antibodies can still exist in patients with SARD, such as SLE (7, 10, 23), and their clinical significance remains to be elucidated. In the present study, anti-DFS70 antibodies seemed to have a certain prevalence in patients who have been diagnosed with SLE and might be associated with anti-dsDNA.

In our study, the prevalence of anti-DFS70 antibodies in SLE patients (20.7%) was higher than that in age- and gender-matched HC (9.5%) and DC (10.8%) groups. When comparing with different studies (**Supplementary Table S4**), we found that the rate of anti-DFS70-positive patients in the SLE cohort was beyond most previous studies, while close to the research based on Asian SLE patients by Hayashi et al. (11) and Kang et al. (24), showing 22.1% and 15.7% of anti-DFS70 prevalence, separately. In addition to the effects of detection methods, the discrepancy in the prevalence of anti-DFS70 antibodies may result from genetic, ethnic, and environmental factors. A multicenter study that enrolled 1,137 SLE patients from different countries revealed that Canadian and European patients were less likely to have anti-DFS70 antibodies than patients residing in the USA and Asia (9). Although the prevalence of anti-DFS70 antibodies in SLE patients in our study differed from other studies, the frequency of monospecific anti-DFS70 antibodies was still low at 1.2% (10/851), which is consistent with the previously reported rate (0.4%–3.1%) (9, 12, 16). Moreover, the prevalence of anti-DFS70 antibodies (9.5%) in Chinese healthy individuals was at a low level within the previously reported range (8.9%–33.3%) of healthy individuals (12, 13, 16).

Except for the prevalence, there were some interesting findings in clinical associations of anti-DFS70 antibodies. The anti-DFS70-positive SLE patients were associated with younger age, which corresponded with the study of Watanabe et al. (6) that showed a significantly higher positive rate of anti-DFS70 antibodies in hospital staff under 35 years than those 35 years and older. Moreover, a close relationship between anti-DFS70 and anti-dsDNA was verified by both the stepwise multivariable logistic regression model and Spearman’s rank correlation test. In this study, anti-DFS70-positive SLE patients were associated with a higher frequency of anti-dsDNA, and the positive correlations between titers of anti-DFS70 and anti-dsDNA antibodies were observed as well. It was consistent with our recent study, which suggested that anti-DFS70 antibodies were associated with anti-dsDNA antibodies in lupus nephritis (LN) patients (15). In addition, our findings also agree with one recent study on anti-DFS70 antibodies that showed that all eight anti-DFS70-positive patients with SARD were Chinese and 75% (6/8) of them had anti-dsDNA antibodies as well (25). It indicated a potential relationship of anti-DFS70 antibodies with anti-dsDNA in Chinese SLE patients.

While comparing the association between anti-DFS70 antibodies and clinical indicators in SLE patients, there were some differences between our research and published studies (**Table 4**; **Supplementary Table S5**). We found significant associations of anti-DFS70-positive SLE patients with a higher frequency of anti-dsDNA and anti-C1q, more rapid ESR, and lower concentrations of C3, which are related to active SLE; however, our findings were distinct from the listed three studies (8, 11, 14) in **Table 4**. Choi et al. and Aragón et al. reported negative associations between anti-DFS70 and anti-dsDNA, and Mahler et al. did not find any association between these two parameters. These differences seemed to be controversial, but they might be due to several reasons, such as different populations, ethics, detection methods, and even the disease status of patients, which needed to be elucidated by further investigation. Moreover, we observed a significant association of anti-DFS70 antibodies with mucosal ulcers by multivariable analysis, which has not been reported before (**Supplementary Table S5**). Mucosal ulcers were a common manifestation reflecting the dermal inflammatory process of SLE (26). Moreover, anti-DFS70 antibodies were reported to commonly exist in various inflammatory diseases such as alopecia, atopic dermatitis, and idiopathic uveitis (10), which indicated that the presence of anti-DFS70 antibodies may be a reflex of inflammatory status.

**Table 4:** Comparison of demographic and serological features between anti-DFS70-positive and anti-DFS70-negative adult SLE patients in the present study versus other referral SLE cohorts.

	Present study	Mahler et al. (12)	Choi et al. (9)	Aragón et al. (16)
Country of residence	China	Canada	Canada, USA, Mexico, UK, Iceland, Sweden, Scotland UK, Spain, Denmark, Turkey, Korea	Colombia
SLE sample size	851	251	1,137	64
Prevalence of anti-DFS70 (%)	20.7^a^	2.8^b^	7.1^b^	12.5^c^
Prevalence of monospecific anti-DFS70 (%)	1.2^a^	0.4^b^	1.1^b^	3.1^c^
Demographic features				
** Age**	*↓	NS	NS	/
Sex	NS	NS	NS	/
Disease duration	NS	NS	NS	/
SLEDAI-2K	NS	NS^d^	NS	NS^d^
Serology				
** Anti-dsDNA**	***↑	NS	S↓	S↓
** Anti-C1q**	***↑	NS	/	/
Anti-Sm	NS	NS	NS	NS
Anti-nRNP/Sm	*↑	NS	S↓	NS
Anti-Ro52	NS	/	NS	/
Anti-SSA/Ro60	NS	NS	S↓	NS
Anti-SSB/La	NS	NS	S↓	NS
** Anti-PCNA**	***↑	/	NS	/
Anti-Rib-P	NS	/	NS	/
Anticardiolipin	NS	/	/	NS
** C3**	***↓	/	/	***↑
CRP	NS	/	/	NS
** ESR**	**↑	/	/	*↓

Boldfaced characters are the events showing non-uniform tendencies in various studies.

C3, complement 3; CRP, C-reactive protein; DFS70, dense fine speckles 70; ds-DNA, double-stranded DNA; ESR, erythrocyte sedimentation rate; PCNA, proliferating cell nuclear antigen; Rib-P, ribosomal-P; SLE, systemic lupus erythematosus; SLEDAI, Systemic Lupus Erythematosus Disease Activity Index; SLEDAI-2K, Systemic Lupus Erythematosus Disease Activity Index 2000; “↑” positively associated to patients with anti-DFS70 antibodies; “↓” negatively associated to patients with anti-DFS70 antibodies; “/” no data; NS, no significance; S, statistical significance (specific p-value was not reported).

^*^p < 0.05; ^**^p < 0.01; ^***^p < 0.001.

a Anti-DFS70 antibodies were measured by enzyme-linked immunosorbent assay.

b Anti-DFS70 antibodies were measured by chemiluminescent immunoassay.

c Anti-DFS70 antibodies were measured by immunofluorescence immunoadsorption.

d Study used SLEDAI score instead of SLEDAI-2K score.

In the long-term follow-up, we found a positive correlation between anti-dsDNA titers with both the O.D. value of anti-DFS70 by in-house ELISA and the anti-DFS70 index by commercial LIA kits. Furthermore, patients in the anti-DFS70 changed group who experienced decreasing results of anti-DFS70 antibodies from the first visit to the last visit showed significantly decreased titers of anti-dsDNA during the same period, which emphasized the close relationship between anti-DFS70 and anti-dsDNA. In addition, by evaluating the dynamic change of anti-DFS70 antibodies with markers of disease activity for 12 anti-DFS70 titers changed follow-up patients (**Figure 4**), a similar variation trend was observed between anti-DFS70 and anti-dsDNA in 83.3% (10/12) of them. All the data from the follow-up group advocated the consistency of dynamically changing between anti-DFS70 and anti-dsDNA antibodies.

Since anti-dsDNA antibodies have been regarded as one of the most important serological SLE biomarkers measured longitudinally in routine clinical practice for the assessment of disease activity (27), we once hypothesized a potential relationship of anti-DFS70 antibodies with disease activity in SLE. However, both the logistic regression and correlation analysis did not show a significant association between anti-DFS70 and SLEDAI-2K. Moreover, some researchers are concerned about the function of the DFS70/LEDGFp75 protein as a DNA binding transcription co-activator and the potential impact of its multiple DNA and chromatin binding domains on assay performance. They considered that the strong affinity of full-length DFS70/LEDGFp75 antigen for DNA/chromatin may raise the possibility to promote indirect interactions mediated by circulating immune complexes in the sera and combination with other indirect dsDNA-based interactions. This might yield increased false-positive results of anti-DFS70 reactivity (28). Therefore, we performed a DNA adsorption test to determine whether the anti-DFS70 reactivity was influenced by the accompanied anti-dsDNA antibodies in our study (**Supplementary Table S3**). In both anti-DFS70 (+) and anti-dsDNA (+) positive serum samples, the anti-DFS70 O.D. values were nearly unchanged after the anti-dsDNA antibodies were adsorbed by DNA, indicating that the anti-DFS70 reactivity was independent of anti-dsDNA antibodies in SLE patients. Given that anti-dsDNA antibodies have been involved in tissue inflammation and damage of SLE patients (29, 30), and that the potential relevance of anti-DFS70 antibodies with inflammatory conditions was suggested by present and previous studies, the mechanism of a positive association of anti-DFS70 antibodies with anti-dsDNA in SLE patients warrants further investigation.

The positive correlations of anti-C1q antibody levels with anti-DFS70 ELISA O.D. value (*r* = 0.181, *p* < 0.0001) in the SLE cohort and with the LIA index (*r* = 0.202, *p* = 0.014) in the follow-up group were observed, separately. However, there was no significant association of anti-C1q frequency with anti-DFS70 antibodies by multivariable logistic regression analysis in 851 SLE patients, which corresponded with our recent study based on LN patients (15).

The limitations of this study include the following: the follow-up was not conducted from the first time each patient was diagnosed with SLE, and follow-up patients did not have the same time points for assessment, which might hinder us from thoroughly assessing the role of anti-DFS70 on a long-term basis. Despite these limitations, our research studied the anti-DFS70 antibodies in a large Chinese SLE cohort and compared them with age- and gender-matched HC as well as DC. We were the first to evaluate the variations of anti-DFS70 antibodies in SLE patients *via* a long-term follow-up.

In conclusion, this study revealed that anti-DFS70 antibodies were more prevalent in Chinese SLE patients than in healthy individuals and other SARD. We found a positive association of anti-DFS70 with anti-dsDNA antibodies in both cross-sectional and long-term follow-up SLE patients, and the latter further indicated a consistent dynamic variation between the levels of anti-DFS70 and anti-dsDNA antibodies. This study provides new perspectives for clinicians to reconsider the role of anti-DFS70 antibodies in SLE patients and the potential links between anti-DFS70 and anti-dsDNA antibodies.

## Data availability statement

The raw data supporting the conclusions of this article will be made available by the authors, without undue reservation.

## Ethics statement

The studies involving human participants were reviewed and approved by Institutional Review Board of Renji Hospital.Written informed consent for participation was not required because: Serum samples were obtained from the residual samples in Clinical Laboratory Department, the requirement for informed consent was waived.

## Author contributions

YD, EL, DC, XN, and ZW collected serum samples and clinical data from patients. YD, DC, and EL performed the experiments. YD, LL, and BZ analyzed and interpreted the data. YD and BZ wrote the manuscript. LL and BZ supervised the study and revised the manuscript. All authors contributed to the article and approved the submitted version.

## Funding

This work was supported by the Zhongnanshan Medical Foundation of Guangdong Province (ZNSXS-20220007), the National Key Research and Development Program of China (Grant No. 2017YFC0909002), the National Natural Science Foundation of China (Grant No. 81974251), and Shanghai Sailing Program (22YF1438400).

## Acknowledgments

The authors would like to thank Dr. Min Li and Dr. Qian Liu (Renji Hospital, Shanghai Jiao Tong University School of Medicine) for their technical support.

## Conflict of interest

The authors declare that the research was conducted in the absence of any commercial or financial relationships that could be construed as a potential conflict of interest.

## Publisher’s note

All claims expressed in this article are solely those of the authors and do not necessarily represent those of their affiliated organizations, or those of the publisher, the editors and the reviewers. Any product that may be evaluated in this article, or claim that may be made by its manufacturer, is not guaranteed or endorsed by the publisher.
