# Supplementary material for: Anti-DFS70 antibodies in systemic lupus erythematosus: Prevalence in a large Chinese cohort and an unexpected association with anti-dsDNA antibodies by a long-term follow-up
Source: Front Immunol. 2022 Sep 14;13:913714. doi: 10.3389/fimmu.2022.913714 (PMC9515321; doi:10.3389/fimmu.2022.913714)
Supplement: Supplementary file 1 [file DataSheet_1.docx]

Supplementary Material

**Supplementary Figure 1. Correlations and coincidence between anti-DFS70 O.D. value by ELISA and the anti-DFS70 index by LIA in 149 samples of follow-up SLE patients.** DFS70, dense fine speckles 70; ELISA, enzyme-linked immunosorbent assay; LIA, line immunoassay; *r*, correlation coefficient; ELISA (-), O.D. value < 0.6, negative; ELISA (+), O.D. value ≥ 0.6, positive; LIA (-), index 0-0.79, negative; LIA (o), index 0.8-1.14, borderline; LIA (+), index ≥ 1.15, positive. LIA (o) was considered as negative, and the coincidence rate between ELISA and LIA was 87.2% (130/149).

**Supplementary Table 1**. Patient characteristics and clinical features of SLE patients with isolated anti-DFS70 antibodies

| **Patient ID** | **Sex** | **Age, yrs** | **Disease duration, yrs** | **SLEDAI-2K** | **Clinical manifestations in the descriptors of SLEDAI-2K** |
| --- | --- | --- | --- | --- | --- |
| 61 | Female | 34 | 3 | 2 | Low complement |
| 116 | Female | 40 | 0.08 | 2 | Pleurisy |
| 176 | Female | 54 | 20 | 4 | Proteinuria |
| 203 | Female | 47 | 0.08 | 11 | Haematuria, Pyuria, Low complement, Thrombocytopenia |
| 557 | Female | 35 | 12 | 11 | Arthritis, Pyuria, Pericarditis, Thrombocytopenia |
| 792 | Female | 65 | 2 | 1 | Fever |
| 828 | Female | 20 | 2 | 14 | Haematuria, Proteinuria, Pyuria, Low complement |
| 834 | Female | 54 | 0.67 | 0 | - |
| 837 | Female | 45 | 3 | 0 | - |
| 839 | Female | 48 | 9 | 8 | Proteinuria, Pyuria |

-, Not have any following clinical manifestations: seizures, psychosis, organic brain syndrome, visual disturbance, cranial nerve disorder, lupus headache, cerebrovascular accident, vasculitis, arthritis, myositis, urinary casts, hematuria, proteinuria, pyuria, pleurisy, pericarditis, rash, alopecia, mucosal ulcers, fever, thrombocytopenia, leukopenia;

DFS70, dense fine speckles 70; SLE, systemic lupus erythematosus; SLEDAI-2K, Systemic Lupus Erythematosus Disease Activity Index 2000.

**Supplementary Table 2**. Comparison of clinical features and symptoms between anti-DFS70-positive and anti-DFS70-negative SLE patients

| **Clinical features and symptoms** | **Anti-DFS70 positive, n=176**  **n (%)** | **Anti-DFS70 negative, n=675**  **n (%)** | **Univariable analysis** | |  | **Multivariable analysis** | |
| --- | --- | --- | --- | --- | --- | --- | --- |
|  |  |  | **Unadjusted OR**  **(95% CI)** | ***P* value** |  | **Adjusted OR**  **(95% CI)** | ***P* value** |
| Neurological | 5 (2.8) | 18 (2.7) | 1.067 (0.391, 2.916) | 0.899 |  |  |  |
| Musculoskeletal | 10 (5.7) | 45 (6.7) | 0.843 (0.416, 1.709) | 0.636 |  |  |  |
| Renal | 105 (59.7) | 378 (56.0) | 1.162 (0.829, 1.628) | 0.383 |  |  |  |
| Serositis | 31 (17.6) | 162 (24.0) | 0.677 (0.442, 1.037) | 0.073 |  |  |  |
| Vasculitis | 0 (0) | 0 (0) | N/A | N/A |  |  |  |
| Rash | 19 (10.8) | 70 (10.4) | 1.046 (0.612, 1.789) | 0.870 |  |  |  |
| Alopecia | 4 (2.3) | 6 (0.9) | 2.593 (0.724, 9.291) | 0.143 |  |  |  |
| Mucosal ulcers | 6 (3.4) | 4 (0.6) | 5.921 (1.652, 21.215) | **0.006** |  | 5.921 (1.652, 21.215) | **0.006** |
| Fever | 17 (9.7) | 46 (6.8) | 1.462 (0.816, 2.619) | 0.202 |  |  |  |
| Thrombocytopenia | 39 (22.2) | 131 (19.4) | 1.182 (0.790, 1.770) | 0.416 |  |  |  |
| Leukopenia | 28 (15.9) | 71 (10.5) | 1.609 (1.003, 2.583) | **0.049** |  |  |  |

DFS70, dense fine speckles 70; N/A, not applicable; Musculoskeletal items: arthritis, myositis; Neurological items: seizures, psychosis, organic brain syndrome, visual disturbance, cranial nerve disorder, lupus headache, cerebrovascular accident; OR, odds ratio; Renal items: urinary casts, hematuria, proteinuria, pyuria; Serositis items: pleurisy, pericarditis; SLE, systemic lupus erythematosus; SLEDAI-2K, Systemic Lupus Erythematosus Disease Activity Index 2000; *P* < 0.05 is shown in bold.

**Supplementary Table 3. Anti-dsDNA titers and anti-DFS70 O.D. values of samples in DNA adsorption test**

| **Test samples** | **Anti-dsDNA (IU/ml)** | |  | **Anti-DFS70 (O.D. value)** | |
| --- | --- | --- | --- | --- | --- |
|  | **No adsorption** | **Adsorption** |  | **No adsorption** | **Adsorption** |
| Anti-DFS70 (+) /Anti-dsDNA (+) | | | | | |
| 1 | 720.51 (+) | 88.92 (-) |  | 1.000 (+) | 0.904 (+) |
| 2 | 783.16 (+) | 352.18 (+) |  | 1.100 (+) | 1.114 (+) |
| 3 | 856.13 (+) | 523.37 (+) |  | 1.354 (+) | 1.325 (+) |
| 4 | 622.44 (+) | 46.87 (-) |  | 0.756 (+) | 0.667 (+) |
| 5 | 713.80 (+) | 282.82 (+) |  | 1.102 (+) | 1.086 (+) |
| 6 | 737.86 (+) | 508.93 (+) |  | 2.088 (+) | 2.060 (+) |
| Anti-DFS70 (-) /Anti-dsDNA (+) | | | | | |
| 7 | 527.78 (+) | 24.18 (-) |  | 0.284 (-) | 0.275 (-) |
| 8 | 851.72 (+) | 539.00 (+) |  | 0.389 (-) | 0.378 (-) |
| 9 | 587.51 (+) | 187.00 (+) |  | 0.402 (-) | 0.445 (-) |
| 10 | 395.07 (+) | 65.52 (-) |  | 0.327 (-) | 0.295 (-) |
| Anti-DFS70 (+) /Anti-dsDNA (-) | |  |  |  |  |
| 11 | 36.66 (-) | 15.14 (-) |  | 0.878 (+) | 0.731 (+) |
| 12 | 41.55 (-) | 7.07 (-) |  | 1.252 (+) | 1.035 (+) |
| 13 | 40.57 (-) | 9.02 (-) |  | 1.458 (+) | 1.434 (+) |

DFS70, dense fine speckles 70; O.D. value, optical density value;

anti-dsDNA reactivity: (+), positive, anti-dsDNA titer ≥ 100 IU/ml; (-), negative, anti-dsDNA titer＜100 IU/ml; anti-DFS70 reactivity: (+), positive, anti-DFS70 O.D. value ≥ 0.6; (-), negative, anti-DFS70 O.D. value＜0.6.

**Supplementary Table 4**. Population and prevalence of anti-DFS70 antibodies in SLE patients in different studies

| **Population** | **Continent** | **Country** | **Sample size of SLE cohorts** | **Prevalence of anti-DFS70, n (%), methodology** | **References** |
| --- | --- | --- | --- | --- | --- |
|  |  |  |  |  |  |
| SLE cohorts | North America, Europe, Asia | Canada, USA, Mexico, UK, Iceland, Sweden, Scotland UK, Spain, Denmark, Turkey, Korea | 1137 | 81 (7.1), CIA | Choi et al. [1] |
|  | North America | Canada | 251 | 7 (2.8), CIA | Mahler et al. [2] |
|  |  | Canada | 24 | 0 (0), CIA | Fitch-Rogalsky et al. [3] |
|  |  | USA | 51 | 1 (2.0), ELISA | Dai L et al. [4] |
|  |  | USA | 36 | 0 (0), IB |  |
|  | Latin America | Colombia | 64 | 8 (12.5), IFA immunoadsorption | Aragón et al. [5] |
|  | South America | Brazil | 87 | 0 (0), IFA, IB | Mariz et al. [6] |
|  | Europe | Italy | 31 | 0 (0), CIA | Bizzaro et al. [7] |
|  |  | Denmark | 131 | 3 (2.3), CIA | Nilsson et al. [8] |
|  | Asia | China | 851 | 176 (20.7), ELISA | **Present study** |
|  |  | China | 377 | 74 (19.6), ELISA | Chen D et al. [9] |
|  |  |  | 268 | 53 (19.8), ELISA |  |
|  |  | Japan | 68 | 15 (22.1), ELISA | Hayashi et al. [10] |
|  |  |  |  | 3 (4.4), IFA |  |
|  |  | Japan | 124 | 7 (5.64), IB | Muro et al. [11] |
|  |  | Japan | 55 | 1 (1.8), IFA, IB | Watanabe et al. [12] |
|  |  | Korea | 13 | 2 (15.4), IFA | Kang et al. [13] |
|  |  | Turkey | 101 | 3 (2.97), IFA | Peker et al. [14] |
|  |  |  |  | 3 (2.97), ELISA |  |
|  |  | Israel | 33 | 1 (3.03), CIA | Shovman et al. [15] |
|  |  |  |  | 1 (3.03), IFA |  |

CIA, chemiluminescent immunoassay; DFS70, dense fine speckles 70; ELISA, enzyme-linked immunosorbent assay; IB, immunoblotting; IFA, indirect immunofluorescence assay; LIA, line immunoblot assay; SLE, systemic lupus erythematosus; SLEDAI-2K, Systemic Lupus Erythematosus Disease Activity Index 2000.

**Supplementary Table 5**. Comparison of clinical features and symptoms between anti-DFS70-positive and anti-DFS70-negative SLE patients in present study versus other referral SLE cohorts

|  | **Present study** | | **Mahler et al. [2]** | **Choi et al. [1]** | **Aragón et al. [5]** |
| --- | --- | --- | --- | --- | --- |
| SLEDAI-2K | | NS | NS† | NS | NS† |
| Neurological | | NS | NS | NS | NS |
| **Musculoskeletal** | | NS | NS | S↑ | NS |
| Renal | | NS | NS | NS | NS |
| Serositis | | NS | NS | NS | NS |
| Vasculitis | | N/A | / | NS | / |
| Rash | | NS | NS | NS | / |
| Alopecia | | NS | / | NS | / |
| **Mucosal ulcers** | | **↑ | NS | NS | / |
| Fever | | NS | / | NS | / |
| Thrombocytopenia | | NS | NS | NS | NS |
| **Leukopenia** | | *↑ | NS | NS | NS |

DFS70, dense fine speckles 70; Musculoskeletal items: arthritis, myositis; Neurological items: seizures, psychosis, organic brain syndrome, visual disturbance, cranial nerve disorder, lupus headache, cerebrovascular accident; Renal items: urinary casts, hematuria, proteinuria, pyuria; Serositis items: pleurisy, pericarditis; SLE, systemic lupus erythematosus; SLEDAI, systemic lupus erythematosus disease activity index; SLEDAI-2K, Systemic Lupus Erythematosus Disease Activity Index 2000; † Study used SLEDAI score instead of SLEDAI-2K score; * *P* < 0.05, ** *P* < 0.01, *** *P* < 0.001; ↑ Positively associated to patients with anti-DFS70 antibodies positive; /, No data; N/A, not applicable; NS, no significance; S, statistical significance (specific *P* value was not reported); Boldfaced characters were the events showed ununiform tendencies in various researches.

**REFERENCE**

1. Choi MY, Clarke AE, St Pierre Y, Hanly JG, Urowitz MB, Romero-Diaz J et al. The prevalence and determinants of anti-DFS70 autoantibodies in an international inception cohort of systemic lupus erythematosus patients. Lupus 2017; 26: 1051-1059. doi: 10.1177/0961203317692437

2. Mahler M, Parker T, Peebles CL, Andrade LE, Swart A, Carbone Y et al. Anti-DFS70/LEDGF antibodies are more prevalent in healthy individuals compared to patients with systemic autoimmune rheumatic diseases. J Rheumatol 2012; 39: 2104-2110. doi: 10.3899/jrheum.120598. Epub 2012 Sep 1

3. Fitch-Rogalsky C, Steber W, Mahler M, Lupton T, Martin L, Barr SG et al. Clinical and serological features of patients referred through a rheumatology triage system because of positive antinuclear antibodies. PLoS One 2014; 9: e93812. doi: 10.1371/journal.pone.0093812

4. Dai L, Li J, Ortega R, Qian W, Casiano CA, Zhang JY. Preferential autoimmune response in prostate cancer to cyclin B1 in a panel of tumor-associated antigens. J Immunol Res 2014; 2014: 827827. doi: 10.1155/2014/827827

5. Aragon CC, Posso-Osorio I, Puerta G, Gonzalez JD, Naranjo JC, Echeverri A et al. Prevalence of anti-DFS70 autoantibodies in a Latin American cohort of patients with systemic lupus erythematosus and without autoimmune diseases. Clin Rheumatol 2020; 39: 2163-2169. doi: 10.1007/s10067-020-04990-z

6. Mariz HA, Sato EI, Barbosa SH, Rodrigues SH, Dellavance A, Andrade LE. Pattern on the antinuclear antibody-HEp-2 test is a critical parameter for discriminating antinuclear antibody-positive healthy individuals and patients with autoimmune rheumatic diseases. Arthritis Rheum 2011; 63: 191-200.

7. Bizzaro N, Tonutti E, Tampoia M, Infantino M, Cucchiaro F, Pesente F et al. Specific chemoluminescence and immunoasdorption tests for anti-DFS70 antibodies avoid false positive results by indirect immunofluorescence. Clin Chim Acta 2015; 451: 271-277. doi: 10.1002/art.30084

8. Nilsson AC, Voss A, Lillevang ST. DFS70 Autoantibodies are Rare in Healthy Danish Individuals but may Still Serve as a Diagnostic Aid. Scand J Immunol 2015; 82: 547-548. doi: 10.1111/sji.12366

9. Chen D, Zhao L, Dai Y, Du F, Li E, Niu X et al. Anti-DFS70 Antibodies Are Associated With Proliferative Lupus Nephritis and Renal Pathological Activity. Front Immunol (2022) 13: 810639. doi: 10.3389/fimmu.2022.810639

10. Hayashi N, Uto K, Imanishi A, Sugiyama D, Morinobu A, Saegusa J. Prevalence of anti-dense fine speckled 70 antibodies in healthy individuals and patients with antinuclear antibody-associated autoimmune rheumatic diseases in Japan. Medicine (Baltimore) 2021; 100: e24556. doi: 10.1097/MD.0000000000024556

11. Muro Y, Sugiura K, Morita Y, Tomita Y. High concomitance of disease marker autoantibodies in anti-DFS70/LEDGF autoantibody-positive patients with autoimmune rheumatic disease. Lupus 2008; 17: 171-176.

12. Watanabe A, Kodera M, Sugiura K, Usuda T, Tan EM, Takasaki Y et al. Anti-DFS70 antibodies in 597 healthy hospital workers. Arthritis Rheum 2004; 50: 892-900. doi: 10.1002/art.20096

13. Kang SY, Lee WI. Clinical significance of dense fine speckled pattern in anti-nuclear antibody test using indirect immunofluorescence method. Korean J Lab Med 2009; 29: 145-151. doi: 10.3343/kjlm.2009.29.2.145

14. Peker BO, Sener AG, Tarhan EF, Kaya S. Investigation of anti-DFS70 antibody in patients with systemic autoimmune rheumatic diseases. Clin Rheumatol 2019; 38: 3627-3633. doi: 10.1007/s10067-019-04730-y

15. Shovman O, Gilburd B, Chayat C, Amital H, Langevitz P, Watad A et al. Prevalence of anti-DFS70 antibodies in patients with and without systemic autoimmune rheumatic diseases. Clin Exp Rheumatol 2018; 36: 121-126.
