# Supplementary figures and images for: Anti-DFS70 antibodies in systemic lupus erythematosus: Prevalence in a large Chinese cohort and an unexpected association with anti-dsDNA antibodies by a long-term follow-up
Source: Front Immunol. 2022 Sep 14;13:913714. doi: 10.3389/fimmu.2022.913714 (PMC9515321; doi:10.3389/fimmu.2022.913714)

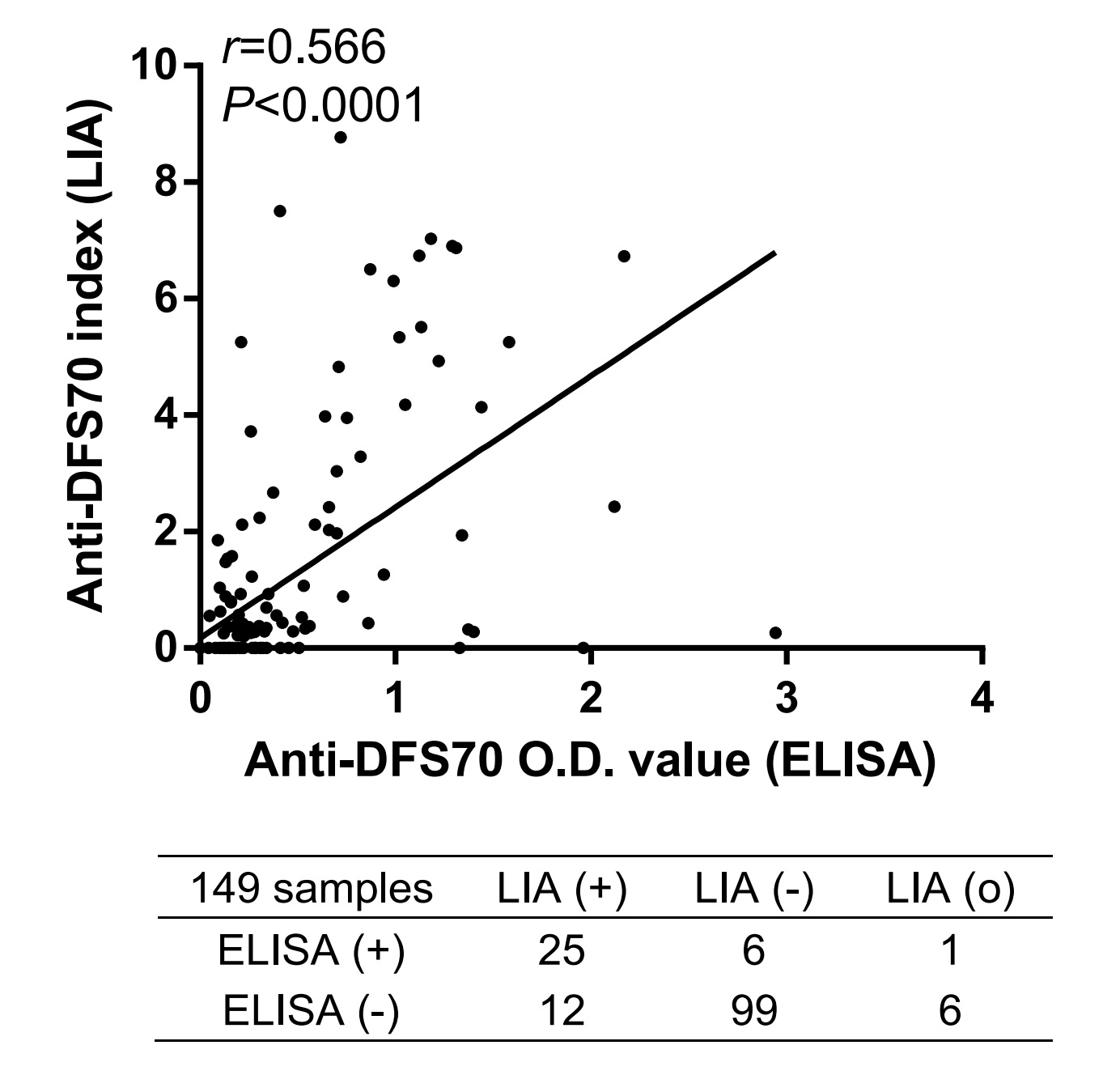

Supplement: Supplementary file 2 [file Image_1.jpeg]
